# The expression and roles of parent-of-origin genes in early embryogenesis of angiosperms

**DOI:** 10.3389/fpls.2014.00729

**Published:** 2014-12-16

**Authors:** An Luo, Ce Shi, Liyao Zhang, Meng-Xiang Sun

**Affiliations:** ^1^State Key Laboratory of Hybrid Rice, Department of Cell and Developmental Biology, College of Life Sciences, Wuhan UniversityWuhan, China; ^2^College of Life Sciences, Yangtze UniversityJingzhou, China

**Keywords:** uniparental transcripts, gamete-delivered transcript, maternal control, paternal allele, genomic imprinting, embryo

## Abstract

Uniparental transcripts during embryogenesis may arise due to gamete delivery during fertilization or genomic imprinting. Such transcripts have been found in a number of plant species and appear critical for the early development of embryo or endosperm in seeds. Although the regulatory expression mechanism and function of these genes in embryogenesis require further elucidation, recent studies suggest stage-specific and highly dynamic features that might be essential for critical developmental events such as zygotic division and cell fate determination during embryogenesis. Here, we summarize the current work in this field and discuss future research directions.

## INTRODUCTION

**FIGURE 1:**
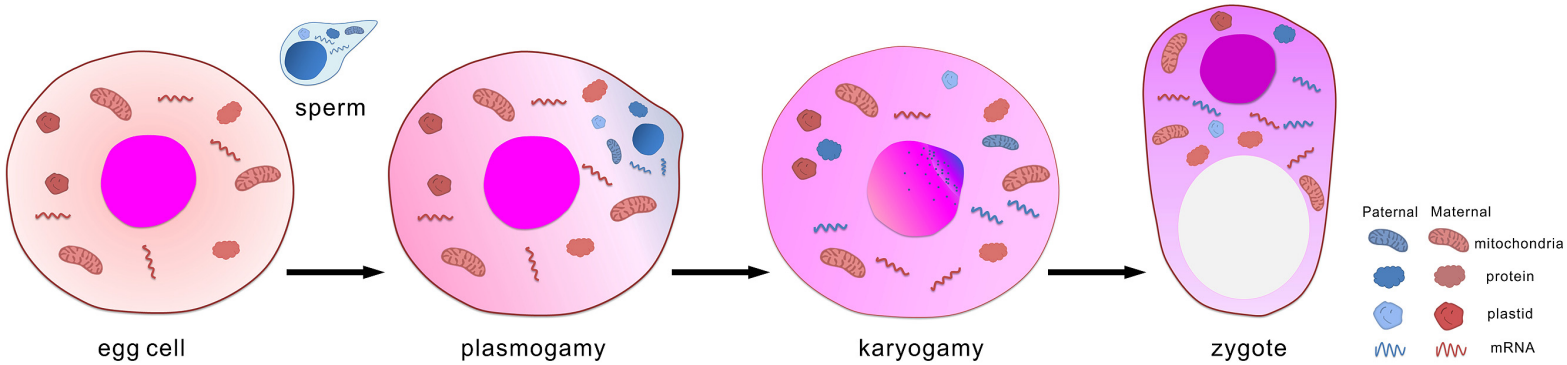
**Fertilization integrates the maternal and paternal genetic information**.

During sexual reproduction of flowering plants, male and female gametes are formed in the haploid gametophytic generation (Walbot and Evans, 2003; Chang et al., 2011). In angiosperm, the typical female gametophyte contains two kinds of female gametes, a haploid egg cell and a diploid central cell with two identical copies of the maternal genome. The male gametophyte is found in pollen, which carries one generative cell or two sperm cells. During pollen generation, sperm cells are transported though the pollen tube to the female gametophyte. Upon double fertilization, two sperm cells enter the embryo sac. One sperm cell fuses with the egg cell, and the other fuses with the central cell. This integration of the two gamete genomes results in the formation of a diploid embryo and a triploid endosperm, respectively. After fertilization, the embryo forms basic morphological and physiological structures (Le et al., 2007), during which the endosperm plays a nutritive role, similar to the placenta of mammals, to support embryonic development (Lopes and Larkins, 1993; Olsen, 2004). During this process, both paternal and maternal genetic information may contribute to the fertilization and development of the embryo, which leads to generation of the sporophyte. These parental information includes RNA that transcribed in sperm and/or egg cells, proteins that synthesized and deposited in gametes, paternal or maternal genome, and mitochondria and plastid genome. After fertilization they are brought into and integrated in zygote (**Figure 1**). Due to technical limitations the contribution of gamete-delivered proteins, mitochondria and plastid genome to zygote development and early embryogenesis are hardly investigated. Current studies as pioneer works mainly focus on *de novo* expression of imprinted genes and gamete-delivered transcripts.

The molecular mechanisms of fertilization and early embryogenesis, especially the role of parent-of-origin genes, have been well studied in animals. However, little is known about these processes in plants due to technical limitations. Gametogenesis, fertilization and embryogenesis occur deep in the plant saprophytic tissues, thus rendering it difficult to observe the developmental events and investigate the molecular mechanisms of these processes directly. Modern technological advances have allowed the isolation and analysis of gametes, zygotes, and early embryos in a wide variety of plants including maize, tobacco, *Arabidopsis*, rice, and wheat (Engel et al., 2003; Zhao et al., 2011; Nodine and Bartel, 2012; Anderson et al., 2013; Domoki et al., 2013). Therefore, great advances have been made toward understanding the role of uniparental transcripts in plant embryogenesis.

In animals, maternal allele products synthesized during gametogenesis exert control in all aspects of embryonic development prior to the global activation of the zygotic genome (Tadros and Lipshitz, 2009). However, in plants, the parental contribution in early embryogenesis has not yet been fully understood. Early reports indicated that the transcripts in early embryos were mainly originated from the maternally inherited alleles and the transcription of paternal alleles was delayed (Vielle-Calzada et al., 2000; Baroux et al., 2001; Golden et al., 2002; Grimanelli et al., 2005). Baroux et al. (2008) further suggested that early embryogenesis in plants was maternally controlled similar to that in animals, as early studies indicated that maternal transcripts could support embryonic development until the proembryo stage. At the same time, some other researches presented evidences of early activated paternal genome (Weijers et al., 2001; Scholten et al., 2002; Lukowitz et al., 2004; Sheldon et al., 2008). Recently, paternal transcripts were proved to be critical for the normal development of early embryo (Ueda et al., 2011; Babu et al., 2013). More impressively, interleukin-1 receptor-associated kinase (IRAK)/Pelle-like kinase gene, *SHORT SUSPENSOR (SSP)* transcripts were found to be produced in mature pollen and were believed to be carried into the egg cell via fertilization in *Arabidopsis thaliana* (Bayer et al., 2009). *SSP* functioned during the asymmetric first division in the zygote, indicating that the paternal transcripts from sperm cells may be involved in many aspects of zygotic development and early embryogenesis in plants. Our previous work also confirmed that paternal transcripts in sperm cells could be found in zygotes soon after fertilization (Xin et al., 2011), suggesting the possibility that sperm-delivered paternal transcripts may be involved in zygotic development.

Meyer and Scholten (2007) reported the relative expression levels of parental transcripts in zygotes, suggesting equivalent parental contribution in maize zygotic development. Maternally expressed in embryo 1 *(mee1)* in maize was the first reported imprinted gene in a plant embryo, although its function is unclear (Jahnke and Scholten, 2009). Using deep sequencing in a genome-wide analysis, Autran et al. (2011) assessed the parental contributions in early embryogenesis and found that the maternal transcripts predominated at early embryonic stages in *Arabidopsis*. With development, the relative paternal contribution arose due to the gradual activation of the embryonic genome. Subsequently, Nodine and Bartel (2012) found that a majority of genes were expressed equally from both parents at the beginning of embryogenesis in *Arabidopsis*. Interestingly, some of these works focused on the quantitative ratio of maternal and paternal transcripts, some mainly analyzed the regulatory roles of these genes. It is not surprised to see various conclusions. Even more, a latest report indicated that the different results might be due to the different material (e.g., ecotypes) they used in their experiments (Del Toro-De León et al., 2014). Despite all these discussions, it is believed that some transcripts are derived primarily from one parent or from imprinted genes in embryos soon after fertilization (Autran et al., 2011; Nodine and Bartel, 2012). These studies indicate that the parent-of-origin gene transcripts indeed exist in the zygote or early embryo. Such transcripts could arise from both the gamete-delivered and *de novo* expression of imprinted genes. Each type of uniparental transcript may play specific roles in plant development, since they are regulated by different molecular mechanisms. This review highlights the characteristics of uniparental transcripts during early embryogenesis.

## GAMETE-CARRIED MATERNAL OR PATERNAL TRANSCRIPTS INVOLVED IN EARLY EMBRYOGENESIS

### MATERNAL TRANSCRIPTS

The embryo originates from a fertilized egg cell, termed a zygote. Two sequential events occur during the integration of a sperm and an egg cell: plasmogamy and karyogamy. Not only do the two genomes integrate, but also various components of the cytoplasm mix during the fertilization process. For example, sperm mitochondria could be found in fertilized egg cells of tobacco (Yu and Russell, 1994) although mitochondria is usually inherited maternally.

During early embryogenesis in most animal species, maternal transcripts deposited in the egg cells are involved in various developmental processes before activation of the zygotic genome, such as formation of embryonic axes, cell differentiation, and morphogenesis (Johnston, 1995; Wylie et al., 1996; Nishida, 1997; Angerer and Angerer, 2000; Mohr et al., 2001; Pellettieri and Seydoux, 2002). Although various experimental data support the hypothesis that maternal control may also exist during early embryogenesis in plants (Baroux et al., 2008), little is known about transcripts stored in egg cells and their role in early embryogenesis (Xin et al., 2012).

Using microdissection, Sprunck et al. (2005) constructed a cDNA library from wheat egg cells, and a total of 404 clusters were found to function in metabolic activity, mRNA translation and protein turnover. Subsequently, another 226 expressed sequence tags (ESTs) were studied in wheat egg cells (Domoki et al., 2013). In a similar analysis carried out in tobacco, thousands of ESTs were detected, which may be involved in a variety of developmental processes (Ning et al., 2006; Zhao et al., 2011). In addition, microarray technology combined with laser-assisted microdissection (LAM) was used to analyze the expression profile in *Arabidopsis* egg cells (Wuest et al., 2010). Transcriptomic analysis of egg cells isolated by manual manipulation was performed in rice (Ohnishi et al., 2011; Abiko et al., 2013), and genome-wide deep sequencing was used to characterize the gene expression profile in rice egg cells (Anderson et al., 2013). The functional categories of approximately 27,000 genes detected proved to be comprehensive. However, a comparison of the egg-specific expression of transcriptomes in rice and *Arabidopsis* revealed relatively different sets of genes in egg cells of rice and *Arabidopsis* (Ohnishi et al., 2011).

The role of mRNA stored in egg cells has been investigated. Although downregulation of RNA polymerase II by RNA interference (RNAi) impeded *de novo* transcription, the development of *Arabidopsis* embryos continued until the preglobular stage (Pillot et al., 2010). In tobacco, zygotic development continued without *de novo* transcription until 72 h after pollination (HAP). The cytological observation of developmental events in transcriptionally inhibited zygotes showed that maternal transcripts stored in egg cells were functionally competent in gamete fusion, zygote volume reduction, complete cell wall formation, large vacuole disappearance, and limited cell enlargement during early developmental stages. However, *de novo* transcripts would then seize control of embryogenesis to trigger subsequent developmental processes (Zhao et al., 2011).

Interestingly, small RNA-mediated transposon silencing is thought to be an essential regulatory mechanism in male and female gametes (Slotkin et al., 2009; Martínez and Slotkin, 2012). Anderson et al. (2013) evaluated the expression of genes involved in the miRNA and siRNA pathways in transcriptomes of rice gametes and showed that all important components involved in these pathways were active in egg cells rather than in sperm cells. Thus, transposon silencing is mediated by small RNAs produced in egg cells; moreover, it is regulated in the zygote by small RNAs inherited from the egg cells (Anderson et al., 2013).

Currently, the roles of female gamete transcripts in zygotes and early embryogenesis are unclear. Although *de novo* transcription in the zygotic genome is activated within hours after fertilization in maize, tobacco and *Arabidopsis*, the maternal transcripts deposited in the egg cells still play a key role in the initial stages of zygotic development (Meyer and Scholten, 2007; Zhao et al., 2011; Nodine and Bartel, 2012).

### PATERNAL TRANSCRIPTS

The sperm cell, the other contributor to zygote, has a simple structure including the karyoplasm and very little cytoplasm. Due to the condensed chromatin observed in sperm cells, it was generally thought that inactive male transcription made no contribution to early embryogenesis prior to zygotic genome activation. This view might be supported in animals, since almost all mRNAs in zygotes are inherited from egg cells (Ostermeier et al., 2004; Krawetz, 2005). However, the cytoplasm of sperm cells may play an important role during early embryogenesis after fertilization in plants, as the extracted sperm nuclei in maize was insufficient to achieve successful fertilization *in vitro* (Matthys-Rochon et al., 1994).

Recently, increasing evidence has confirmed the presence of a number of transcripts in the sperm cell, refuting the hypothesis that highly condensed chromatin in sperm cells impede activation of transcription. Various cDNA libraries have been constructed based on isolated sperm cells from rice (Gou et al., 2001), tobacco (Xin et al., 2011), maize (*Zea mays*; Engel et al., 2003), and Plumbago dimorphic (Gou et al., 2009). Additionally, genome-wide expression has been detected in different plants. Using microarray analysis, the transcriptomic profile in sperm cells was investigated in rice and *Arabidopsis* (Borges et al., 2008; Russell et al., 2012; Abiko et al., 2013). The transcriptomes of rice sperm cells were studied by deep sequencing (Anderson et al., 2013), and ~25000 genes were analyzed. These studies revealed a diverse and broad constitution of mRNAs in sperm cells. Subsequently, sperm transcription profiles were compared among different plant species. Only 35 genes were found in common among 1,048 ESTs in tobacco (Xin et al., 2011), 5,829 genes in *Arabidopsis* (Borges et al., 2008), and 5,174 ESTs in maize (Engel et al., 2003). Analysis of these 35 genes suggested that active transcription in sperm cells is involved in many basic pathways and processes such as metabolism, transcription, translation, signal transduction and intercellular trafficking (Xin et al., 2011).

For years, plant scientists have questioned whether male transcripts are delivered to the zygote during the fertilization process, and if so, whether these sperm-carrying transcripts have a role in zygote development or early embryogenesis. In our previous work, we identified sperm-specific transcripts in zygotes at 96 HAP (Ning et al., 2006). Subsequently, two kinds of sperm transcripts with unknown function were revealed in zygotes ~10 h after fertilization (HAF). These results strongly suggested that paternal transcripts could be delivered into zygotes, where they might play a role in zygote activation and/or early embryogenesis (Xin et al., 2011). Similarly, Ohnishi et al. (2014) found abundant expression of the Os07g0182900 rice gene in sperm cells (Abiko et al., 2013), but not in unfertilized egg cells. The fact that its transcripts could be detected in the zygote ~10–20 min after fertilization indicated that transcripts in plant zygotes could be delivered from the sperm cells by plasmogamy. The Os07g0182900 gene encoding cytosine-5 DNA methyltransferase 1 (MET1) may be involved in the transition from the zygote to two-celled pre-embryo stage, as the process could be partially inhibited by a specific inhibitor of MET1 (Abiko et al., 2013). In *Arabidopsis*, the polarity of elongated zygotes contributed substantially to regular embryonic development. Corrected asymmetric cell division led to normal formation of the initial apical–basal axis and the embryo and suspensor ancestors in plants (Jeong et al., 2011; Zhang and Laux, 2011; Ueda and Laux, 2012). Sperm transcripts are now believed to be essential in this critical developmental process. Bayer et al. (2009) reported that transcripts of the IRAK/Pelle-like kinase gene, *SSP,* were produced in mature sperm cells and translated in zygotes after fertilization. Defective *SSP* influenced the elongation of the zygote and the formation of suspensor through the YODA-dependent MAPKKK signaling pathway. These examples suggest that sperm mRNAs might have vital functions in normal developmental embryogenesis.

## IMPRINTED GENES IN EARLY EMBRYOGENESIS

In mammals and flowering plants, genomic imprinting is a general epigenetic mechanism associated with the differential expression of parental alleles (Feil and Berger, 2007). The differential *de novo* transcription of parental alleles is caused by different epigenetic influences established in the germ line, rather than the nucleotide changes or uniparental transcripts caused by gamete delivery. Maternally expressed imprinted genes (MEGs) are expressed maternally but silenced paternally, whereas maternally expressed imprinted genes (PEGs) are expressed paternally but silenced maternally.

### IMPRINTED GENES IN PLANT EMBRYOS

Imprinting is another cause of unequal contributions from parental transcripts in the early embryo. A minority of imprinted genes has been identified in endosperm using conventional methods, such as sequence homologies, small-scale transcriptional surveys, assays for reduced DNA methylation and mutant identification. The *mee1* gene in maize provided the evidence confirming the presence of imprinted genes in embryos (Raissig et al., 2011). The differential methylation status between paternal and maternal alleles regulates the maternal expression of *mee1* in the embryo and endosperm. Dynamic expression of *mee1* was found in the early embryo, but its function remains unclear (Jahnke and Scholten, 2009).

Recently, genome-wide approaches have been used to identify imprinted genes in *Arabidopsis*, maize and rice (Gehring et al., 2011; Hsieh et al., 2011; Luo et al., 2011; Waters et al., 2011; Wolff et al., 2011; Zhang et al., 2011). Several 100 endosperm-specific imprinted genes were newly detected in these species. However, the presence of imprinted genes in the embryo remains controversial. For example, Hsieh et al. (2011) identified 116 MEGs and 10 PEGs in *Arabidopsis* endosperm 7–8 days after pollination (DAP), while 37 MEGs and one PEG were found in the embryo during the same period. However, the imprinted genes in the embryo were considered to be false positives due to contamination with endosperm or maternal tissue (Hsieh et al., 2011). Similarly, Gehring et al. (2011) identified 165 MEGs and 43 PEGs in *Arabidopsis* endosperm at 6–7 DAP; additionally, 17 MEGs and one PEG were found in embryos during the same period. However, the imprinted genes in the embryo could have been due to endosperm contamination or biased expression dependent on an unchangeable allele (Gehring et al., 2011). In monocots, Luo et al. (2011) found 262 imprinted loci in rice endosperm at 5 DAF. An imprinted gene was detected in both the embryo and endosperm; however, this candidate requires further confirmation by confirming its expression in gametes (Luo et al., 2011). Waters et al. (2011) found 54 MEGs and 46 PEGs in maize endosperm at 14 DAP, with 29 MEGs and nine PEGs in embryos during the same period. However, these imprinted genes in embryos might be due to contamination, trafficking of transcripts produced in the endosperm to the embryo, or relatively stable transcripts inherited from the gametes (Waters et al., 2011).

Currently, genomic imprinting in *Arabidopsis* embryos has not been validated conclusively. Raissig et al. (2013) constructed cDNA libraries using 2 to 4-cells embryos and globular embryos isolated from the reciprocal cross of the Col-0 and the Ler accessions. Imprinted gene candidates were then chosen, and their relative expression levels between parental alleles were assessed by reverse transcription polymerase chain reaction (RT-PCR) and Sanger sequencing (Raissig et al., 2013). A total of 11 MEGs were expressed at the 2 to 4-cells and globular embryo stages, and one PEG was expressed at the 2 to 4-cells embryo stage. No transcripts in the one PEG or in nine of the MEGs were detected in the gametes, indicating that their imprinted expression in the embryo was derived from *de novo* transcription and was reliable. To avoid contamination, strict procedures were adopted in constructing the cDNA libraries. In addition, an independent assay was used to confirm the genomic imprinting in embryos by fusing the promoters of seven MEGs and one PEG with the reporter gene β-glucuronidase (GUS). Promoter-GUS reporter lines (Col-0 background) were crossed reciprocally with wild-type plants (Col-0), and the analysis of stained F1 embryos showed that six MEG reporter lines were either imprinted fully or showed a strong bias for maternal expression (Raissig et al., 2013). Furthermore, Raissig et al. (2013) detected imprinted expression of all embryonic MEGs and the PEG in other samples, as early Col-0 × Cvi embryos (different accession, similar stage; Nodine and Bartel, 2012) and late torpedo-stage Col-0 × Ler embryos (same accessions, but later stage; Gehring et al., 2011). The results confirmed that the expression of most imprinted genes during early embryogenesis was maintained regardless of the different accessions or later developmental stage (Raissig et al., 2013). Therefore, these results indicated that genomic imprinting may not be restricted to the endosperm and may be more extensive in embryos than thought previously.

### FUNCTION OF IMPRINTED GENES INVOLVED IN EMBRYOGENESIS

In mammals, 100s of imprinted genes have been identified that are connected to the location of nutrient transfer from mother to offspring, embryogenesis, and postnatal development (Constância et al., 2004; Gregg et al., 2010). Abnormal imprinting can harm fetal growth, hormone systems after birth, and adult brain function. Whereas genome-wide approaches have revealed many imprinted genes involved in transcriptional regulation, chromatin modification, hormone signaling, ubiquitin degradation, small RNA pathways and metabolism (Gehring et al., 2011; Hsieh et al., 2011; Luo et al., 2011; Raissig et al., 2011, 2013) in plants, little is known regarding the involvement of imprinted genes in plant development (**Table 1**).

**Table 1:** Uniparental genes in sexual plant reproduction.

	Genes	Epigenetic mark	Ecotype/inbred line for detection	Period of detection	Mutant/RNAi phenotype	Reference
**Endosperm**
*Arabidopsis*	*MEA* (MEG)^1,2^	H3K27me3 DNA-me	L*er*&RLD	6,7,8 DAP	Seed abortion	Kinoshita et al. (1999), Vielle-Calzada et al. (1999)
	*FWA* (MEG)	DNA-me	Col-0, Ler, WS	6,7,8 DAP	—	Kinoshita et al. (2004),
						Jullien et al. (2006)
	*FIS2* (MEG)^1^	DNA-me	C24&Col	0.5-5 DAP	Seed abortion	Jullien et al. (2006, 2008),
						Luo et al. (2000)
	*MPC* (MEG)^1^	DNA-me	Col&L*er*	3,5,7 DAP	Abnormal seed	Tiwari et al. (2008)
	*PHE1* (PEG)^1,2^	H3K27me3	Col&C24	1–4 DAP	—	Köhler et al. (2003, 2005),
		DNA-me				Makarevich et al. (2008)
	*FH5* (MEG)^1,2^	H3K27me3	L*er*&C24	5 DAP	Endosperm defects	Ingouff et al. (2005), Fitz Gerald et al. (2009)
	*AGL36* (MEG)^1^	H3K27me3 DNA-me	Col&Ler	3 DAP	—	Shirzadi et al. (2011), Wuest et al. (2010)
	3 MEGs&2 PEGs	DNA-me	Col-*gl*&L*er*	Torpedo-stage	—	Gehring et al. (2009)
	116 MEGs&10 PEGs	—	Col&L*er*	7–8 DAP	—	Hsieh et al. (2011)
	165 MEGs&43 PEGs	—	Col-0&Ler	6 or 7 DAP	—	Gehring et al. (2011)
	39 MEGs&27 PEGs	—	Col-0&Bur-0	4DAP	—	Wolff et al. (2011)

Maize	*Fie1* (MEG)^2^	DNA-me,	B73&Mo17;	2-15 DAP	—	Danilevskaya et al. (2003),
		H3K27me3,	SSS1&NSS1			Gutiérrez-Marcos et al. (2006),
		H3/H4-Ac				Hermon et al. (2007)
	*Fie2* (MEG)	DNA-me	B73&Mo17	2,5 DAP	—	Danilevskaya et al. (2003),
						Gutiérrez-Marcos et al. (2006),
						Hermon et al. (2007),
	*Nrp1* (MEG)^2^	DNA-me,	B73&Mo17;	10,14,21 DAP	—	Guo et al. (2003),
		H3K27me3,	SSS1&NSS1			Haun and Springer (2008)
		H3/H4-Ac				
	*Peg1* (PEG)	—	W22&Tx303	12 DAP	—	Gutiérrez-Marcos et al. (2003)
	*Meg1* (MEG)^1,2^	DNA-me	F2, A69Y, W23	4 DAP	Reduced-size seeds	Gutiérrez-Marcos et al. (2004), Costa et al. (2012)
	*Mez1* (MEG)	DNA-me,	B73&Mo17	8-27 DAP	—	Haun et al. (2007)
		H3K27me3,				Haun and Springer (2008)
		H3/H4-Ac				
	*Mee1* (MEG)	DNA-me	UH005&UH301	6 DAP	—	Jahnke and Scholten (2009)
	*α-Tubulin* (MEG)	DNA-me	W64A&A69Y	20 DAP	—	Lund et al. (1995b)
	*Zein* (MEG)	DNA-me	W64A&A69Y	19 DAP	—	Lund et al. (1995a)
	*R gene* (MEG)	—	—	—	—	Kermicle (1970), Ludwig et al. (1989)
	*Dzr-1* (MEG)	—	BSSS53&Mo17	15-27 DAP	—	Chaudhuri and Messing (1994)
	54 MEGs&46 PEGs	—	B73&Mo17	14 DAP	—	Waters et al. (2011)
	93 MNCs&124 PNCs^3^	—	B73&Mo17	10DAP	—	Zhang et al. (2011)

Rice	177 MEGs&85 PEGs	—	Nip&93-11	5 DAF	—	Luo et al. (2011)

**Embryo**
*Arabidopsis*	11 MEGs&1 PEGs^1,2^	H3K27me3^4^	Col-0&Ler	2.5,4 DAP	—	Raissig et al. (2013)
Maize	*Mee1* (MEG)^2^	DNA-me	UH005&UH301	3,6,8 DAP	—	Jahnke and Scholten (2009)

	**Gamete-carried transcripts**		**Origin of transcripts**		**Defective in embryogenesis**	**Reference**

**Zygote**
*Arabidopsis*	*SSP*		Sperm-specific		Abnormal division of zygote	Bayer et al. (2009)
Rice	*Os07g0182900*		Sperm-specific		Abnormal division of zygote	Ohnishi et al. (2014)
Tobacco	*Ntsp0002*&*Ntsp0003*		Sperm-specific		—	Xin et al. (2011)

*The table lists the known information of imprinted and potentially imprinted genes discovered in plants to date. Phenotypes in seed development are also displayed. These data are partially adapted from Raissig et al. (2011). —: not shown or not known.*

*^1^Reporter activity of parent-of-origin–expression was identified (part of imprinted genes in *Arabidopsis* embryo).*

*^2^*De novo* transcription was identified (part of imprinted genes in *Arabidopsis* embryo).*

*^3^MNCs, maternal expressed transcripts; PNCs, paternal expressed transcripts. MNCs and PNCs include protein-coding genes and long non-coding RNAs.*

*^4^Epigenetic marks of a part of imprinted genes in *Arabidopsis* embryo.*

To date, only four imprinted genes in endosperm are known to be involved in embryogenesis (Raissig et al., 2011; Costa et al., 2012). In *Arabidopsis*, the FERTILIZATION-INDEPENDENT SEED *(FIS)* genes *MEA* (*FIS1*) and *FIS2* belong to the Polycomb group family (PcG). *MEDEA (MEA)* is expressed in both the embryo and endosperm; however, maternal imprinting has been confirmed only in the latter, and it remains to be determined whether *MEA* is imprinted in the embryo (Raissig et al., 2013). *FIS2* is a maternally imprinted gene, and its expression was detected both in the central cell before fertilization and endosperm after fertilization (Luo et al., 2000). Double fertilization products that contained maternal alleles of *mea* and *fis2* resulted in failure of endosperm cellularization. Moreover, embryogenesis ceased at the heart/torpedo stage, resulting in seed abortion (Luo et al., 2000). Another novel maternal imprinted gene MPC was found to be active in the central cell before fertilization and in the endosperm from fertilization to 4 DAP. Knockdown of *MPC* through RNAi resulted in defective seed development, with delayed embryogenesis and abnormal embryo and endosperm morphology (Tiwari et al., 2008). In maize, maternally expressed gene 1 *(Meg1)* encodes a new kind of signaling peptide located in endosperm nutrient transfer cells, where it regulates their establishment and differentiation. *Meg1* is the first identified imprinted gene in plants that participates in nutrient distribution to the embryo. Interestingly, in contrast to imprinted genes in mammals, *Meg1* promotes rather than restricts the transfer of nutrient flow from the mother to fetus (Costa et al., 2012).

The imprinted genes in the endosperm mentioned above have defined roles in the endosperm; however, the role of the imprinted genes in the embryo remain unknown. To identify the contribution of imprinted genes in the embryo during embryogenesis, T-DNA gene insertions were used to search for deviant phenotypes relative to embryonic development, but no obvious phenotypes were observed (Raissig et al., 2013). Interestingly, all the maternally imprinted genes in the *Arabidopsis* embryo were expressed in the seed coat, and some even showed a slightly biased expression toward the basal embryo and the suspensor (Raissig et al., 2013). Notably, some maternally imprinted genes were involved in metabolism (Raissig et al., 2013). Therefore, maternally imprinted genes in the embryo might function at the interface between the embryo and maternal tissue, possibly by linking seed coat metabolism and embryo metabolism, and rendering the genes in the embryo under maternal control (Raissig et al., 2013). This result may support the maternal–offspring coadaptation theory, which posits that maternally imprinted genes are critical for the events during mother–offspring interactions (Bateson, 1994; Wolf and Hager, 2006). Further research on the roles of imprinted genes in the embryo will lead to a better understanding of the function and evolution of genomic imprinting in plants.

### REGULATION OF IMPRINTED GENES IN EMBRYOS

DNA methylation and histone modification are two distinct epigenetic mechanisms involved in the regulation of genomic imprinting in plants. The differential DNA methylation status of parental alleles in the endosperm is due mainly to genome-wide hypomethylation of maternal alleles in the central cell (Gehring et al., 2009, 2011). DNA glycosylase DEMETER *(DME)* with 5-methylcytosine excising activity (Kinoshita et al., 2004; Gehring et al., 2006) and the repression of *MET1* involved in maintaining DNA methylation (Jullien et al., 2008; Hsieh et al., 2011) are responsible for the DNA demethylation at CG sites. However, sometimes DNA methylation alone is not sufficient to establish different imprinting markers of some genes, and the Polycomb repressive complex 2 (PRC2) that catalyzes the trimethylation of histone H3 on lysine 27 (H3K27me3) is required (Baroux et al., 2006; Makarevich et al., 2008; Hennig and Derkacheva, 2009).

To investigate the epigenetic mechanism of genomic imprinting in the embryo, the fertilization-independent endosperm (*fie*) mutant was crossed reciprocally with wild-type plants, and *met1-3* mutants were used to pollinate wild-type plants (Raissig et al., 2013). F1 hybrid embryos were isolated, and mutant embryonic cDNA libraries were created. The detection of the allele-specific expression pattern of 11 embryonic MEGs in *Arabidopsis* demonstrated that imprinted expression of MEGs in embryos are not influenced by the paternal *met1-3* allele. However, disruption of the maternal FIE function changed the monoallelic expression of two MEGs and one PEG. Thus, the function of PRC2 may be comprehensive in regulating imprinted expression in both the embryo and endosperm (Raissig et al., 2013). Furthermore, the role of asymmetric DNA methylation in the CHG context was negated in the establishment of imprinting in the embryo. Finally, Raissig et al. (2013) indicated that PRC2, but not MET1, played a role in regulating the imprinted genes in the embryo. This is consist with a previous conjecture that DNA methylation is unlikely to be a primary imprinting mark in maize embryos (Gutiérrez-Marcos et al., 2006; Jahnke and Scholten, 2009). Other undiscovered mechanisms may be involved in the establishment of genomic imprinting in the embryo.

## PERSPECTIVES

Technological advances in genome-wide sequencing technology and acquisition of gametes, zygotes and early embryos will be important for elucidating the role of parent-of-origin genes during plant early embryogenesis. Such technological progress may lead to the identification of more uniparental transcripts in early embryos, whether gamete-delivered or imprinted gene-derived. Currently, in both gametes and early zygotes, it remains technically difficult to identify the origin of transcripts, which may be gamete-delivered during fertilization or transcribed *de novo* after fertilization. In addition, little is known about the functions of these parent-of-origin genes during fertilization and early embryogenesis. Obviously, no matter how parental transcripts may contribute to the transcriptome of early embryo, the function analysis of the parent-of-origin genes in specific developmental events at specific developmental stage will surely provide imperative knowledge to understand the parental effect in early seed formation.

Recently, the identification of imprinted genes in early embryos in *Arabidopsis* has questioned the concept that imprinted genes are restricted mainly to the endosperm. In light of so many candidate imprinted genes in the embryos of dicots and monocots, optimized methods are required to avoid contamination of maternal tissues and false positives or negatives in data collection and reliable analysis. Determination of the relevant functions of imprinted genes should shed light on our understanding of epigenetic mechanisms in promoting embryogenesis, embryo pattern formation, and cell fate determination during embryogenesis. With further technological advancement, the role of methylation in gene imprinting during embryogenesis might be further elucidated.

## Conflict of Interest Statement

The authors declare that the research was conducted in the absence of any commercial or financial relationships that could be construed as a potential conflict of interest.
